# Experimental Subarachnoid Hemorrhage Drives Catecholamine-Dependent Cardiac and Peripheral Microvascular Dysfunction

**DOI:** 10.3389/fphys.2020.00402

**Published:** 2020-05-13

**Authors:** Danny D. Dinh, Darcy Lidington, Jeffrey T. Kroetsch, Chloe Ng, Hangjun Zhang, Sergei A. Nedospasov, Scott P. Heximer, Steffen-Sebastian Bolz

**Affiliations:** ^1^Department of Physiology, University of Toronto, Toronto, ON, Canada; ^2^Toronto Centre for Microvascular Medicine at The Ted Rogers Centre for Heart Research Translational Biology and Engineering Program, University of Toronto, Toronto, ON, Canada; ^3^Engelhardt Institute of Molecular Biology, Moscow, Russia; ^4^Sirius University of Science and Technology, Sochi, Russia; ^5^Heart & Stroke/Richard Lewar Centre of Excellence for Cardiovascular Research, University of Toronto, Toronto, ON, Canada

**Keywords:** tumor necrosis factor, myogenic response, myocardial stunning, adrenergic signaling, mechanosensor

## Abstract

Subarachnoid hemorrhage (SAH) is a devastating cerebral event caused by an aneurysmal rupture. In addition to neurological injury, SAH has significant effects on cardiac function and the peripheral microcirculation. Since these peripheral complications may exacerbate brain injury, the prevention and management of these peripheral effects are important for improving the overall clinical outcome after SAH. In this investigation, we examined the effects of SAH on cardiac function and vascular reactivity in a well-characterized blood injection model of SAH. Standard echocardiographic and blood pressure measurement procedures were utilized to assess cardiac function and hemodynamic parameters *in vivo*; we utilized a pressure myography approach to assess vascular reactivity in cremaster skeletal muscle resistance arteries *ex vivo.* We observed that elevated catecholamine levels in SAH stun the myocardium, reduce cardiac output and augment myogenic vasoconstriction in isolated cremaster arteries. These cardiac and vascular effects are driven by beta- and alpha-adrenergic receptor signaling, respectively. Clinically utilized adrenergic receptor antagonists can prevent cardiac injury and normalize vascular function. We found that tumor necrosis factor (TNF) gene deletion prevents the augmentation of myogenic reactivity in SAH: since membrane-bound TNF serves as a mechanosensor in the arteries assessed, alpha-adrenergic signaling putatively augments myogenic vasoconstriction by enhancing mechanosensor activity.

## Introduction

Subarachnoid hemorrhage (SAH) is a devastating cerebral event, most frequently caused by an aneurysmal rupture (van Gijn et al., 2007). Although SAH is a cerebrovascular pathology first and foremost, it also inflicts significant damage to peripheral organs, including the heart and vascular system (Chen et al., 2014). These peripheral manifestations are clinically significant, as perturbations in cardiac output and vascular resistance may exacerbate neurological injury and increase mortality (Yarlagadda et al., 2006; van der Bilt et al., 2009): thus, mitigating cardiac injury and effectively manipulating blood pressure are key aspects of SAH therapy. Remarkably, blood pressure management in SAH is a particularly understudied field and consequently, current clinical guidelines (Connolly et al., 2012) are primarily based on small studies, rather than solid, large-cohort evidence (Grise and Adeoye, 2012). Trials testing blood pressure management regimes have not been undertaken, in part, due to the complexity of the task: many molecular mechanisms contribute to blood pressure control, both at the level of the heart and the microcirculation, but their relative contributions in the perturbed state of SAH have not been adequately defined. This makes the safe and reliable manipulation of blood pressure difficult in a patient that is particularly vulnerable.

Sympathetic activity and circulating catecholamines are rapidly and persistently elevated in SAH (Benedict and Loach, 1978; Naredi et al., 2000), forming an intuitive link between the primary injury within the brain and organ injury at remote sites. At the level of the heart, the most frequent neurocardiogenic manifestation in SAH is “stunned myocardium,” a transient deficiency in left ventricular function arising from excessive catecholamine stimulation (Wybraniec et al., 2014; Kerro et al., 2017); in a small subset of patients, a more severe form of catecholamine toxicity materializes as a transient ballooning of the left ventricular apex (takotsubo cardiomyopathy) (Kerro et al., 2017).

Even in the presence of a pathological reduction in cardiac output, elevated blood pressure remains an inescapable clinical feature of SAH (Qureshi et al., 2007). Blood pressure in SAH patients typically follows a biphasic course: initially, there is a rapid rise (within 2 days) that plateaus for 3–6 days; a proportion of patients then subsequently experience a spontaneous secondary increase in blood pressure that strongly associates with the secondary delayed ischemia and poorer neurological outcome (Faust et al., 2014; Fontana et al., 2015). The immediate rise in blood pressure is almost certainly attributable to the elevated circulating catecholamines and sympathetic activity; however, the secondary hypertension that arises later is not associated with an additional elevation in catecholamine levels (Naredi et al., 2000; Fontana et al., 2015): this indicates that a distinct vascular mechanism drives this rise in blood pressure.

The myogenic response is a critical autoregulatory mechanism that prominently contributes to the control of vascular tone and hence, peripheral resistance (Lidington et al., 2013). Although myogenic vasoconstriction is an agonist-independent, mechanosensitive response to transmural pressure, it plays a remarkably important role in amplifying pressor responses (Metting et al., 1989a, b). In fact, myogenic amplification is a larger contributor to the overall blood pressure response (>60%) than the actual stimulus itself (Metting et al., 1989a). Intriguingly, adrenergic signaling has the capability to feed forward and augment myogenic mechanisms (Meininger and Faber, 1991; Ping and Johnson, 1992): thus, sustained catecholamine stimulation may initiate a positive feedback loop that further enhances vasoconstriction in the absence of additional increases in circulating catecholamine levels. This mechanism putatively underlies the secondary, catecholamine-independent increase in blood pressure observed in SAH patients (Naredi et al., 2000; Fontana et al., 2015).

This investigation, therefore, assessed skeletal muscle resistance artery myogenic vasoconstriction in a mouse model of SAH. We selected skeletal muscle resistance arteries for this study, because this vascular bed represents the body’s largest circulatory network (40% of body mass is skeletal muscle) and consequently, myogenic tone in these arteries is a prominent determinant of total peripheral resistance (Korthuis, 2011).

## Materials and Methods

This investigation conforms to the National Research Council’s 2011 *Guide for the Care and Use of Laboratory Animals* (ISBN: 0-309-15400-6). All animal care and experimental protocols were approved by the Institutional Animal Care and Use Committee at the University of Toronto and were conducted in accordance with national animal protection laws.

### Mice

Wild-type mice (C57BL/6N) were purchased from Charles River Laboratories (Montreal, QC, Canada); germ-line tumor necrosis factor (TNF) alpha knockout mice (TNF^–/–^) were purchased from Taconic laboratories (Hudson, NY, United States). Inducible, smooth muscle cell-targeted TNF knockout mice, generated by crossing floxed TNF mice (Grivennikov et al., 2005) with mice expressing a recombinant Cre recombinase under the control of a smooth muscle promoter (SMMHC-CreER^T2^) (Wirth et al., 2008), have been described and utilized in our previous studies (Yagi et al., 2015). Similarly, smooth muscle cell-targeted Gq_11_ knockout mice were generated by crossing floxed Gq_11_ (Gna11) mice (Wettschureck et al., 2001) with SMMHC-CreER^T2^ mice (Wirth et al., 2008) and have been described previously (Althoff et al., 2012). All mice were housed in a controlled climate (21°C, 40–60% humidity) with a standard 14 h:10 h light–dark cycle, fed normal chow and had access to water and food *ad libitum*. All surgical procedures and parameter assessments were conducted during the normal work day, which coincided with the lights-on phase in the light-dark cycle.

### Induction of Subarachnoid Hemorrhage

We used a well-characterized model of experimental SAH (Yagi et al., 2015). Briefly, each mouse was anesthetized (isoflurane) and its head was fixed in a stereotactic frame. A 7 mm incision was made along the midline of the anterior scalp and a 0.9 mm hole drilled into the skull 4.5 mm anterior to the bregma. A spinal needle was advanced to the chiasmatic cistern: 80 μl of arterial blood from an immunologically compatible, syngeneic donor mouse was injected into the intracranial space over 10 s. The injected blood was obtained from a separate wild-type donor mouse, via cardiac puncture, immediately prior to injection and did not contain anticoagulants. Sham-operated animals underwent an identical procedure, with sterile saline injected instead of blood. Following injection, the scalp incision was closed. Sham and SAH mice received buprenorphine (0.05 mg/kg s.c. twice daily for 2 days), with the first dose administered intra-operatively. All experimental measurements were conducted at 2-days post-SAH induction or sham procedure.

### Isolation and Functional Assessment of Resistance Arteries

Mouse cremaster skeletal muscle resistance arteries were dissected from the cremaster muscle, cannulated onto micropipettes, stretched to their *in vivo* lengths and pressurized to 60 mmHg, as previously described (Kroetsch et al., 2017). Mouse olfactory cerebral arteries (a first branch of the anterior cerebral artery) were dissected, cannulated and pressurized to 45 mmHg, as previously described (Yagi et al., 2015). Cremaster and cerebral arteries were imaged with a CCD camera at 40x magnification during myography measurements, with luminal diameter measured using a Crescent Electronics (Windsor, ON, Canada) video edge detector and logged using Photon Technology International FeliX32 analysis software (Horiba Canada, Inc., London, ON, Canada). Myography experiments were conducted in calcium-containing 3-morpholinopropanesulfonic acid (MOPS) buffered saline at 37°C with no perfusion ([mmol/L]: NaCl 147.0, KCl 4.7, CaCl_2_ 1.5, MgSO_4_ 1.2, NaH_2_PO_4_ 1.2, pyruvate 2.0, EDTA 0.02, MOPS 3.0, and glucose 5.0). Vasomotor responses to 10 μmol/L phenylephrine provided an assessment of vessel viability at the beginning of each experiment. Cremaster and cerebral arteries were required to achieve 60 and 30% constriction in response to phenylephrine, respectively: this level approximates 75% of the mean constriction stimulated by 10 μmol/L phenylephrine in the respective artery types.

Myogenic responses were elicited by stepwise 20 mmHg increases in transmural pressure from 20 to 80 mmHg (olfactory arteries) or 100 mmHg (cremaster skeletal muscle resistance arteries). At each pressure step, vessel diameter (dia_active_) was measured once a steady state was reached. Following completion of all dia_active_ measurements, the MOPS buffer was replaced with a Ca^2+^-free version and maximal passive diameter (dia_max_) was recorded at each pressure step.

Calcium sensitivity (i.e., the relationship between microvascular tone and intracellular calcium levels) was assessed by increasing extracellular calcium from 0 to 2 mmol/L under depolarizing conditions; depolarization permits the equilibration of intra- and extra-cellular cellular calcium levels, via the opening of L-type calcium channels. To maintain osmolarity, the NaCl content in the MOPS-buffered saline was reduced to compensate for the changes in osmolarity induced by the altered KCl and CaCl_2_ concentrations. The calcium-free version of this solution is formulated as [mmol/L]: NaCl 26.5, KCl 125.0, MgSO_4_ 1.2, NaH_2_PO_4_ 1.2, pyruvate 2.0, EDTA 1.0, MOPS 3.0, and glucose 5.0. As calcium was added to this solution, NaCl was reduced proportional to the osmotic load of the added calcium (i.e., 1.5 mmol/L of NaCl for 1 mmol/L CaCl_2_).

Myogenic tone was calculated as the percent constriction in relation to the maximal diameter at each respective transmural pressure: tone (% of dia_max_) = [(dia_max_-dia_active_)/dia_max_]x100, where dia_active_ is the vessel diameter in MOPS containing Ca^2+^ and dia_max_ is the diameter in Ca^2+^-free MOPS. Analyses of vasomotor responses to phenylephrine and calcium sensitivity used the same calculation, only in this case, dia_active_ represents the vessel diameter at steady state following application of the given agent.

### Echocardiography

B-mode echocardiographic measurements were collected with a 30 MHz mechanical sector transducer (Vevo 770; Visual Sonics, Toronto, ON, Canada). Systolic and diastolic volumes were calculated using the *area-length single plane* method and were calculated as 8A^2^/3πL, where A is the area of the left ventricle when viewed in B-mode parasternal long axis mode, and L is the length of the left ventricle from the apex to the aortic valve (Tournoux et al., 2011). In subset of experiments, measurements were collected in conjunction with the mean arterial pressure (MAP) measurements, using a Millar SPR-671 micro-tip mouse pressure catheter (Inter V Medical, Inc., Montreal, QC, Canada). Stroke volume (SV), cardiac output (CO), and total peripheral resistance (TPR) were calculated as: SV = diastolic volume – systolic volume; CO = SV x HR; and TPR = MAP/CO.

### Telemetric Blood Pressure Measurement

Continuous MAP measurements were telemetrically collected with a PhysioTel PA-C10 Pressure Transmitter for Mice and Data Exchange Matrix (Data Sciences International, Saint Paul, MN, United States), as previously described (Kroetsch et al., 2017). Briefly, a gel filled catheter, attached to the telemetric blood pressure transducer, was surgically inserted into the right common carotid artery and secured with a silk suture; the transducer was implanted in a subcutaneous pouch on the chest. Mice recovered from the surgical procedure for 7 days prior to collecting baseline measurements.

### ELISA

Plasma epinephrine and norepinephrine levels were measured by ELISA, according to the manufacturer’s instructions (Abnova cat# KA1877; distributed by Cedarlane Laboratories, Burlington, ON, Canada).

### Data Collection and Statistics

A total of 413 mice were used in the present study; only male mice were utilized, as this was a necessary prerequisite for both the experimental model (i.e., cremaster arteries) and smooth muscle cell-targeted gene deletion. Mice were randomized into groups, including treatment interventions, prior to the Sham/SAH surgical procedures; there was no mortality associated with the SAH surgery. Data collection did not require blinding.

All data are expressed as means ± standard error of the mean (SEM), where n is the number of independent measures. Using data from our previous studies (Meissner et al., 2012; Yagi et al., 2015; Kroetsch et al., 2017), we calculated the experimental group sizes required to provide an 80% power level for the detection of the anticipated differences between groups, with a two-tailed alpha level of 0.05. Data were statistically analyzed using GraphPad Prism 8 software (San Diego, CA, United States) using a Shapiro–Wilk normalicy test and parametric comparisons. Specifically, a Student’s *t*-test was utilized for the comparison of two independent groups; for comparison of multiple independent groups, an ANOVA was utilized, followed by a Dunnett’s *post hoc* test. For the comparison of myogenic responses, calcium sensitivity and dose–response relationships, data were analyzed with a two-way ANOVA, followed by a Tukey’s *post hoc* test. In one instance (plasma norepinephrine levels), data were not normally distributed: in this case, a non-parametric Mann–Whitney test was utilized for the statistical comparison; this is noted in the legend. Differences were considered significant at *P* < 0.05.

## Results

### Experimental Subarachnoid Hemorrhage Induces a Cardiovascular Phenotype

**FIGURE 1:**
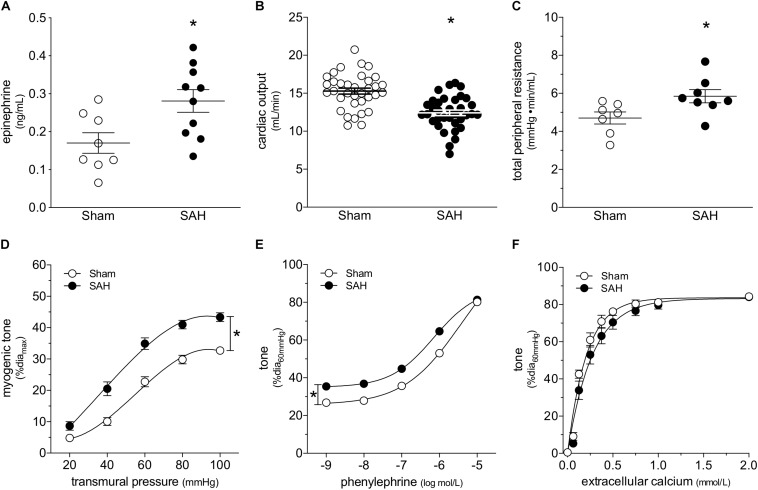
Subarachnoid hemorrhage (SAH) induces a cardiovascular phenotype. At 2-days post-SAH induction, **(A)** plasma epinephrine levels are elevated (sham *n* = 8, SAH *n* = 10), **(B)** cardiac output is reduced (sham *n* = 35, SAH *n* = 35) and **(C)** total peripheral resistance is elevated (sham *n* = 7, SAH *n* = 8). **(D)** The elevated peripheral resistance correlates with augmented cremaster skeletal muscle resistance artery myogenic tone (sham dia_max_: 78 ± 2 μm, *n* = 26; SAH 78 ± 2 μm, *n* = 22). **(E)** Phenylephrine-stimulated vasoconstriction is also greater in cremaster arteries isolated from SAH mice (sham dia_max_: 78 ± 2 μm, *n* = 26; SAH 78 ± 2 μm, *n* = 22); however, **(F)** calcium sensitivity is unaffected (sham dia_max_: 72 ± 5 μm, *n* = 7; SAH dia_max_: 80 ± 3 μm, *n* = 8). *P < 0.05.

At 2-days post-induction, experimental SAH emulates several key peripheral cardiovascular characteristics that are observed clinically, including elevated circulating epinephrine levels (Figure 1A), reduced cardiac output (Figure 1B) and elevated total peripheral resistance (Figure 1C). A summary of the measured echocardiographic parameters can be found in Supplementary Table 1. Although plasma epinephrine levels are elevated, circulating norepinephrine levels are not affected (Supplementary Figure 1). Surprisingly, SAH does not increase MAP at 2-days post-SAH induction (measured under anesthesia; sham = 73 ± 2 mmHg, *n* = 13; SAH = 71 ± 2 mmHg, *n* = 12; P = N.S. by Student’s *t*-test). Consistent with these measures, telemetric blood pressure measurements in conscious mice indicate that SAH does not appreciably alter MAP within 1-week post-SAH induction (Supplementary Figure 2).

At the level of microvascular function, the elevation in total peripheral resistance correlates with augmented cremaster skeletal muscle resistance artery myogenic reactivity (Figure 1D). Representative diameter tracings from vessels isolated from sham and SAH mice are displayed in Supplementary Figure 3. Phenylephrine-stimulated vasoconstriction is also greater in arteries isolated from SAH mice (Figure 1E); however, the upward shift is entirely due to SAH’s effect on myogenic reactivity, which increases basal tone. When baseline tone is normalized, phenylephrine responses in arteries isolated from sham and SAH mice are virtually identical (Supplementary Figure 4). Neither calcium sensitivity (i.e., constriction induced by raising intracellular calcium levels; Figure 1F) nor maximal luminal diameter at 60 mmHg under calcium-free conditions (sham = 78 ± 2 μm, *n* = 26; SAH = 78 ± 2 μm, *n* = 22; P = N.S. by Student’s *t*-test) are affected by SAH. Taken together, the vascular phenotype appears to be specific to the myogenic mechanism.

### β-Adrenergic Receptor Blockade Selectively Normalizes Cardiac Function in Subarachnoid Hemorrhage

**FIGURE 2:**
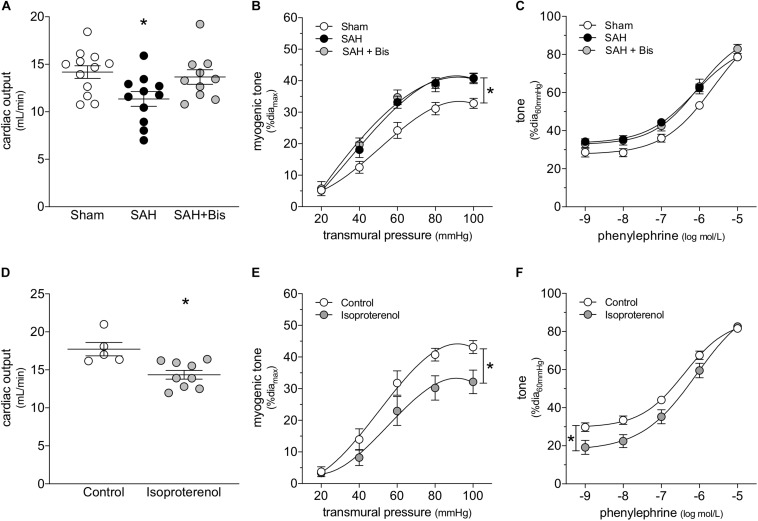
β-Adrenergic signaling drives the cardiac phenotype in SAH, but not the vascular phenotype. **(A)** Bisoprolol (Bis; twice daily for 2 days with i.p. injections; 10 mg/kg initial pre-operative dose followed by 5 mg/kg for all subsequent injections) prevents the compromised cardiac function observed at 2-days post-SAH induction (sham *n* = 12, SAH *n* = 11, SAH + Bis *n* = 10). **(B)** However, bisoprolol treatment does not prevent the augmentation of myogenic reactivity in cremaster skeletal muscle resistance arteries isolated from SAH mice, nor **(C)** does it alter vasoconstriction in response to phenylephrine (sham dia_max_: 80 ± 2 μm, *n* = 13; SAH dia_max_: 80 ± 2 μm, *n* = 10; SAH + Bis dia_max_: 76 ± 2 μm, *n* = 7). **(D)** In naïve mice, the β-adrenergic receptor agonist isoproterenol (150 mg/kg i.p. for 2 days) reduces cardiac output (control *n* = 5, Isoproterenol *n* = 9). However, in contrast to SAH, both **(E)** myogenic reactivity and **(F)** phenylephrine responses in cremaster skeletal muscle resistance arteries isolated from isoproterenol-treated mice are attenuated (control dia_max_: 80 ± 4 μm, *n* = 8; isoproterenol dia_max_: 79 ± 2 μm, *n* = 8). *P < 0.05.

β-Adrenergic receptor blockade is an effective means to prevent catecholamine-induced cardiac injury (Lymperopoulos et al., 2013). In mice with SAH, the β_1_-adrenergic receptor antagonist bisoprolol (mice treated twice daily for 2 days with i.p. injections; 10 mg/kg initial pre-operative dose followed by 5 mg/kg for all subsequent injections) successfully protects cardiac function (Figure 2A); a summary of the measured echocardiographic parameters for bisoprolol-treated SAH mice can be found in Supplementary Table 2. Notably, bisoprolol treatment is only effective when delivered pre-operatively, as we did not observe a protective effect in preliminary experiments when bisoprolol was delivered immediately following the SAH induction surgery (Supplementary Figure 5). This latter finding indicates that: (i) the majority of cardiac injury must occur in the acute phase of SAH, when the catecholamine surge is presumably highest; and (ii) β-blockade can prevent, but not reverse, cardiac dysfunction in SAH. In contrast to its cardioprotective effect, bisoprolol treatment does not prevent the SAH-induced augmentation of cremaster skeletal muscle resistance artery myogenic reactivity (Figure 2B); bisoprolol does not affect phenylephrine responses (Figure 2C) and has no effect in sham mice (Supplementary Figure 6). Consistent with these *in vivo* data, *in vitro* bisoprolol treatment (5 μmol/L, 30 min) does not affect myogenic reactivity or phenylephrine responses in cremaster skeletal muscle resistance arteries isolated from naïve mice (Supplementary Figure 7).

We next treated naïve mice with the β-adrenergic receptor agonist isoproterenol, to determine to what extent selective β-adrenergic receptor activation recapitulates the SAH phenotype. Consistent with our conclusion that β-adrenergic receptors mediate cardiac injury in SAH, isoproterenol (150 mg/kg/day i.p. for 2 days) reduces cardiac output (Figure 2D); a full summary of the measured cardiac and systemic hemodynamic parameters in isoproterenol-treated mice can be found in Supplementary Table 3. Surprisingly, cremaster skeletal muscle resistance artery myogenic reactivity (Figure 2E) and phenylephrine responses (Figure 2F) are both reduced following *in vivo* isoproterenol treatment. In the latter case, the reduced phenylephrine responses are primarily due to a shift in basal myogenic tone: when basal tone is accounted for, phenylephrine responses are not different (Supplementary Figure 8). *In vitro*, isoproterenol (1 μmol/L, 30 min) reproduces the *in vivo* treatment phenotype: reduced myogenic reactivity and phenylephrine responses, with the latter due to reduced myogenic/basal tone (Supplementary Figure 9).

### α-Adrenergic Receptor Blockade Selectively Normalizes Vascular Function in Subarachnoid Hemorrhage

**FIGURE 3:**
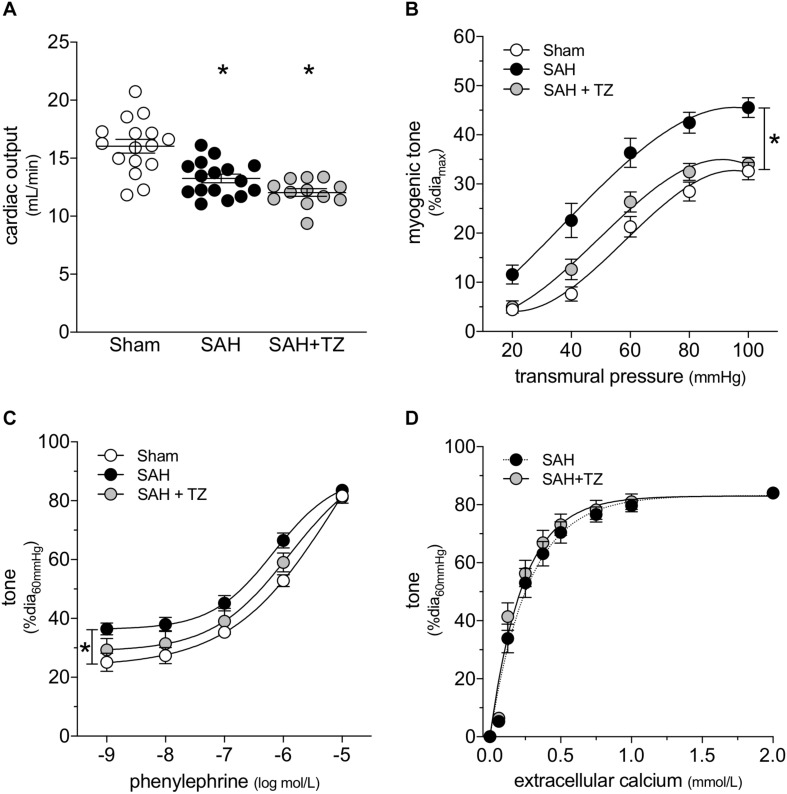
α-Adrenergic signaling drives the vascular phenotype in SAH, but not the cardiac phenotype. **(A)** Terazosin (TZ; twice daily for 2 days with i.p. injections; 1 mg/kg initial pre-operative dose followed by 0.5 mg/kg for all subsequent injections) has no effect on the compromised cardiac function observed at 2-days post-SAH induction (sham *n* = 16, SAH *n* = 16, SAH + TZ *n* = 12). **(B)** However, terazosin treatment successfully normalizes the augmented myogenic reactivity in cremaster skeletal muscle arteries isolated from SAH mice. **(C)** Phenylephrine responses are enhanced in cremaster arteries isolated from SAH mice; this augmentation is also normalized by terazosin treatment (sham dia_max_: 76 ± 3 μm, *n* = 13; SAH dia_max_: 76 ± 3 μm, *n* = 12; SAH + TZ dia_max_: 74 ± 2 μm, *n* = 13). **(D)** Calcium sensitivity is not altered by terazosin treatment (SAH dia_max_: 80 ± 3 μm, *n* = 8; SAH + TZ dia_max_: 78 ± 4 μm, *n* = 7). The SAH curve in **(D)** is reproduced from Figure 1E for comparison to the SAH + TZ curve. *P < 0.05.

As expected, the α_1_-adrenergic receptor antagonist terazosin (mice treated twice daily for 2 days with i.p. injections; 1 mg/kg initial pre-operative dose followed by 0.5 mg/kg for all subsequent injections) does not prevent cardiac dysfunction in mice with SAH (Figure 3A). A summary of the measured echocardiographic parameters for terazosin-treated SAH mice can be found in Supplementary Table 4. Although terazosin is not cardioprotective, it successfully prevents the SAH-induced augmentation of cremaster skeletal muscle resistance artery myogenic reactivity (Figure 3B). In this experimental cohort, phenylephrine responses were statistically higher in arteries isolated from SAH mice, compared to those isolated from sham-operated controls (Figure 3C). The augmented phenylephrine responses are primarily due to a shift in basal tone, as baseline normalization eliminates the difference between the groups (Supplementary Figure 10). This baseline shift is not observed in arteries isolated from terazosin-treated SAH mice and therefore, phenylephrine responses appear normalized in arteries isolated from terazosin-treated SAH mice (Figure 3C). Of note, terazosin also normalizes myogenic reactivity when administered post-SAH induction (Supplementary Figure 11) and therefore, potentially represents a viable treatment option.

As predicted by our Figure 1 data, calcium sensitivity in arteries isolated from terazosin-treated SAH mice is not different from that found in arteries isolated from SAH mice (Figure 3D). Intriguingly, terazosin treatment had a markedly different effect in sham mice, where it augmented myogenic tone (Supplementary Figure 12). *In vitro*, terazosin (25 nmol/L, 30 min) has no effect on myogenic reactivity in cremaster skeletal muscle resistance arteries isolated from naïve mice (Supplementary Figure 13A). As expected, this treatment induces a clear rightward shift in the phenylephrine dose-response relationship (Supplementary Figure 13B; log EC_50_ values for phenylephrine-stimulated vasoconstriction: control = −6.07 ± 0.20, *n* = 6; control + terazosin = −4.91 ± 0.07, *n* = 6; *P* < 0.05 by paired Student’s *t*-test).

**FIGURE 4:**
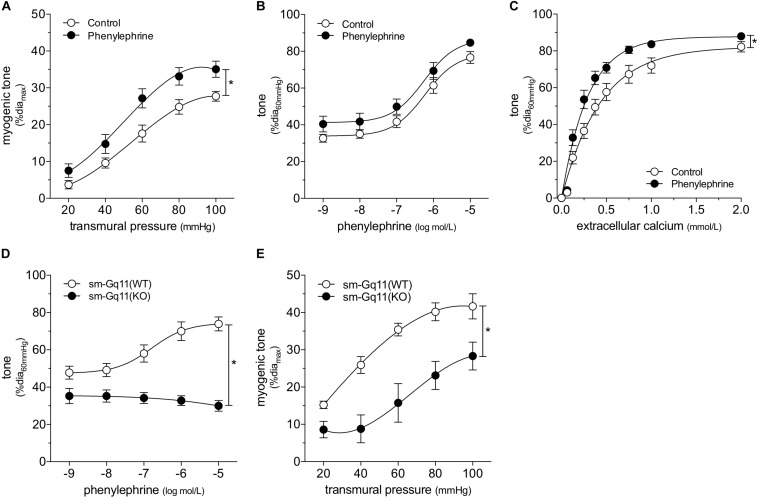
α-Adrenergic signals augment myogenic reactivity *in vitro.* In **(A–C)**, cremaster skeletal muscle resistance arteries isolated from naïve mice were treated for 4 h with either 1 μmol/L phenylephrine or control buffer *in vitro*. The arteries were then washed and assessed in normal buffer. **(A)** Arteries treated with phenylephrine possess higher myogenic reactivity than control arteries. Although *in vitro* phenylephrine treatment **(B)** does not affect subsequent phenylephrine-stimulated vasoconstriction responses, **(C)** calcium sensitivity is increased (control dia_max_: 69 ± 2 μm, *n* = 10; phenylephrine dia_max_: 73 ± 3 μm, *n* = 9). **(D)** Phenylephrine-stimulated vasoconstriction is abolished in cremaster skeletal muscle resistance arteries isolated from smooth muscle cell-targeted Gq_11_ knockout mice [sm-Gq11(KO); dia_max_: 96 ± 10, *n* = 6], relative to wild-type littermate controls [sm-Gq11(WT); dia_max_: 83 ± 6, *n* = 6]. **(E)** In addition to eliminating phenylephrine responses, smooth muscle cell-targeted Gq_11_ gene deletion also attenuates myogenic tone [sm-Gq11(WT) dia_max_: 83 ± 6, *n* = 6; sm-Gq11(KO) dia_max_: 88 ± 12, *n* = 4]. *P < 0.05.

We attempted to replicate the SAH vascular phenotype, by treating cremaster skeletal muscle resistance arteries isolated from naïve mice with phenylephrine *in vitro*. Our preliminary experiments indicated that a relatively long incubation period is required to observe an effect on myogenic tone (*data not shown*). Consistent with our expectations, naïve arteries treated with 1 μmol/L phenylephrine for 4 h display higher myogenic tone than control arteries (Figure 4A); the treatment does not alter phenylephrine-simulated vasoconstriction (Figure 4B). However, in contrast to our sham/SAH data (Figure 1F), calcium sensitivity is higher in phenylephrine-treated arteries, relative to control arteries (Figure 4C). It is notable, however, that the phenylephrine-treated naïve arteries in Figure 4C and the sham-operated arteries in Figure 1F actually have comparable calcium sensitivity (P = N.S. for Figure 1F sham versus Figure 4C phenylephrine; *P* < 0.05 for Figure 1F sham versus Figure 4C control by two-way ANOVA). This profile also applies to myogenic tone at the higher transmural pressures (at both 80 and 100 mmHg, P = N.S. for Figure 1D sham versus Figure 4A phenylephrine; *P* < 0.05 for Figure 1D sham versus Figure 4A control by Student’s *t*-test). Thus, it appears that the phenylephrine treatment maintains the sham level of calcium sensitivity and myogenic tone, while the absence of pro-constrictive signaling over the relatively long incubation period results in reduced calcium sensitivity and myogenic tone. To examine whether non-adrenergic pro-constrictive stimuli also increase calcium sensitivity and myogenic tone *in vitro*, we repeated our long-term incubation experiments using 10 nmol/L angiotensin II. In contrast to phenylephrine (Figure 4), angiotensin II treatment fails to maintain calcium sensitivity and myogenic reactivity (Supplementary Figure 14), despite stimulating vasoconstriction when applied to cremaster arteries.

To further substantiate our conclusion that endogenous catecholamines modulate myogenic reactivity *in vivo*, we assessed myogenic reactivity in cremaster arteries isolated from smooth muscle cell-targeted Gq_11_ knockout mice. Because the loss of Gq_11_ expression abolishes many pro-constrictive signals, including α_1_-adrenergic receptor signaling, this knockout model provides a reasonable *in vivo* recapitulation of prolonged artery incubation in MOPS buffer *in vitro*. As expected, phenylephrine responses are absent in cremaster skeletal muscle resistance arteries isolated from smooth muscle cell-targeted Gq_11_ knockout mice (Figure 4D). Consistent with our hypothesis, myogenic tone is strongly attenuated in arteries isolated from smooth muscle cell-targeted Gq_11_ knockout mice, relative to wild-type controls (Figure 4E).

### Subarachnoid Hemorrhage Augments Myogenic Reactivity by Altering Mechanosensor Function

**FIGURE 5:**
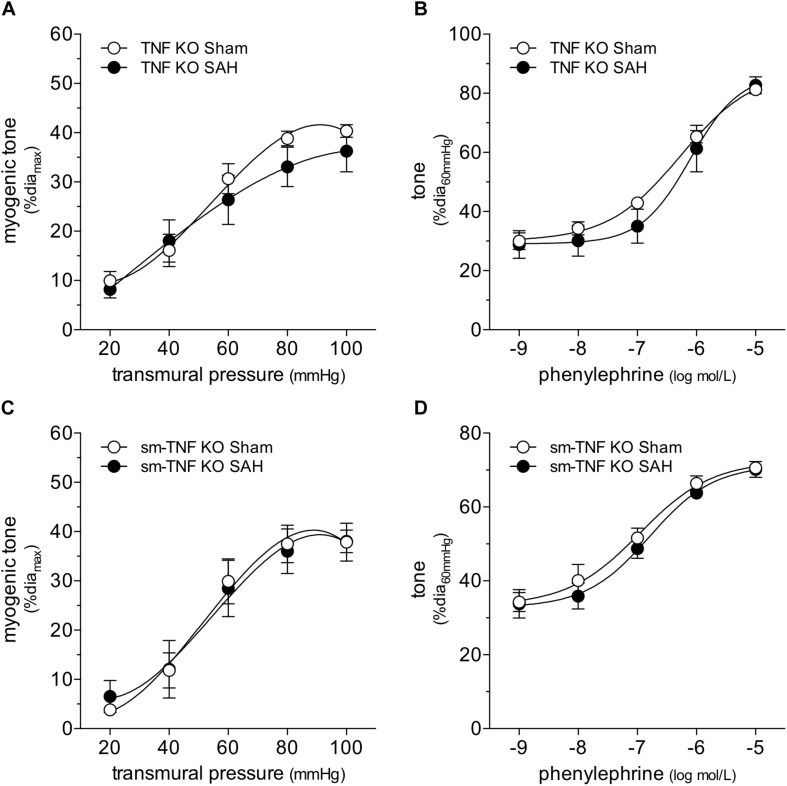
Tumor necrosis factor (TNF) gene deletion abolishes the SAH-induced myogenic tone augmentation. In cremaster skeletal muscle resistance arteries isolated from germline tumor necrosis factor knockout mice (TNF KO), neither **(A)** myogenic tone nor **(B)** phenylephrine responses are affected by SAH (at 2-days post-SAH induction; sham dia_max_: 69 ± 4 μm, *n* = 7; SAH dia_max_: 71 ± 6 μm, *n* = 6). Accordingly, in cremaster skeletal muscle resistance arteries isolated from smooth muscle cell-targeted tumor necrosis factor knockout mice (sm-TNF KO), neither **(C)** myogenic tone (sham dia_max_: 90 ± 4, *n* = 6; SAH dia_max_: 77 ± 7, *n* = 6) nor **(D)** phenylephrine responses are affected by SAH (sham dia_max_: 90 ± 4, *n* = 6; SAH dia_max_: 72 ± 6, *n* = 7).

**FIGURE 6:**
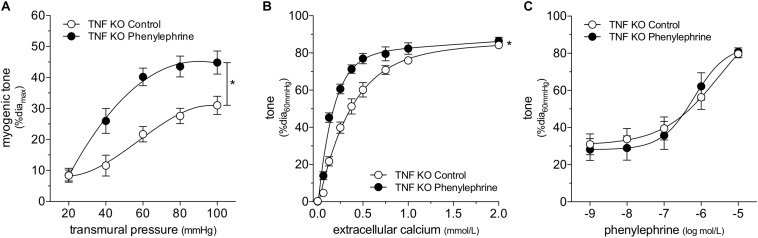
∝-adrenergic signaling augments myogenic reactivity and calcium sensitivity despite tumor necrosis factor (TNF) gene deletion. Cremaster skeletal muscle resistance arteries isolated from TNF KO mice were treated for 4 h with either 1 μmol/L phenylephrine or control buffer *in vitro*. The arteries were then washed and assessed in normal buffer. Arteries treated with phenylephrine display higher **(A)** myogenic reactivity (control dia_max_: 71 ± 4 μm, *n* = 5; phenylephrine dia_max_: 70 ± 3 μm, *n* = 6) and **(B)** calcium sensitivity (control dia_max_: 68 ± 6 μm, *n* = 5; phenylephrine dia_max_: 69 ± 4 μm, *n* = 5) than control arteries; however, the treatment does not affect **(C)** responses to phenylephrine (control dia_max_: 68 ± 6 μm, *n* = 5; phenylephrine dia_max_: 69 ± 4 μm, *n* = 5). *P < 0.05.

Collectively, our data imply that SAH augments myogenic reactivity via an upstream mechanism unique to the myogenic response, rather than by altering general contractility mechanisms that lie downstream. This aspect focused our attention on membrane-bound tumor necrosis factor (mTNF), which serves as the myogenic mechanosensor in cremaster skeletal muscle resistance arteries (Kroetsch et al., 2017). Consistent with our hypothesis that SAH modulates mechanosensor function, SAH fails to augment myogenic tone in cremaster skeletal muscle resistance arteries isolated from both germline and smooth muscle cell-targeted TNF knockout mice (Figure 5). Notably, phenylephrine treatment *in vitro* (1 μmol/L for 4 h) augments myogenic tone and calcium sensitivity in naïve cremaster skeletal muscle resistance arteries isolated from germline TNF knockout mice (Figure 6): thus, the α-adrenergic receptor-dependent effects on calcium sensitivity observed in Figure 4C does not explain the augmented myogenic tone in SAH. In wild-type mice, cremaster skeletal muscle resistance artery TNF mRNA expression does not increase in SAH relative to sham controls (sham = 1.00 ± 0.17 arbitrary units relative to HMBS, *n* = 5; SAH = 0.65 ± 0.21 arbitrary units, *n* = 5; P = N.S. by Student’s *t-*test): this indicates that SAH most likely modulates the mechanosensitive mTNF signaling complex, rather than by solely changing TNF expression itself.

**FIGURE 7:**
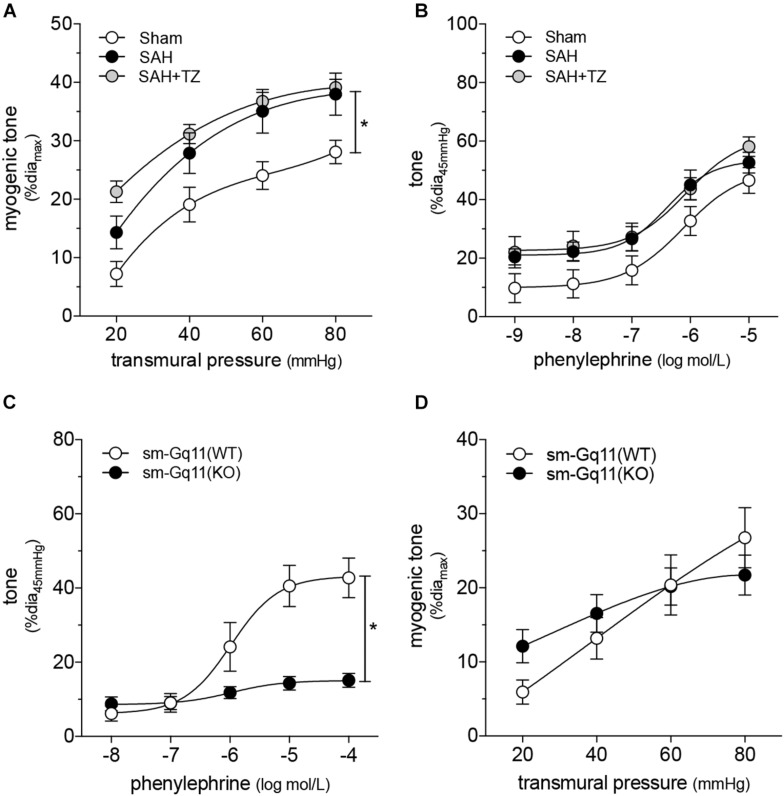
Cerebral and cremaster resistance arteries have distinct phenotypes. **(A)** Terazosin (TZ; twice daily for 2 days with i.p. injections; 1 mg/kg initial pre-operative dose followed by 0.5 mg/kg for all subsequent injections) does not normalize the augmented myogenic reactivity observed in olfactory cerebral arteries isolated from mice at 2-days post-SAH induction (sham dia_max_: 108 ± 4 μm, *n* = 6; SAH dia_max_: 104 ± 7 μm, *n* = 5; SAH + TZ dia_max_: 101 ± 9 μm, *n* = 5). **(B)** Phenylephrine responses in olfactory cerebral arteries are unaffected by SAH or terazosin treatment (sham dia_max_: 110 ± 4 μm, *n* = 5; SAH dia_max_: 104 ± 7 μm, *n* = 5; SAH + TZ dia_max_: 108 ± 10 μm, *n* = 6). **(C)** Phenylephrine-stimulated vasoconstriction is abolished in olfactory cerebral resistance arteries isolated from smooth muscle cell-targeted Gq_11_ knockout mice [sm-Gq11(KO); dia_max_ = 156 ± 5 μm, *n* = 10], relative to wild-type littermate controls [sm-Gq11(WT); dia_max_ = 144 ± 5, *n* = 5]. However, **(D)** there is no effect of the targeted Gq_11_ gene deletion on myogenic tone. *P < 0.05.

To add further credence to our conclusion that SAH modulates mTNF mechanosensor function, we capitalized on a key mechanistic difference between cremaster and cerebral resistance arteries: mTNF does not serve as a mechanosensor in olfactory cerebral arteries (Kroetsch et al., 2017). Consistent with this functional difference, terazosin (mice treated twice daily for 2 days with i.p. injections; 1 mg/kg initial pre-operative dose followed by 0.5 mg/kg for all subsequent injections) fails to normalize the augmented myogenic reactivity in olfactory cerebral arteries (Figure 7A) (Yagi et al., 2015). Phenylephrine responses in olfactory arteries isolated from mice with SAH are not affected by *in vivo* terazosin treatment (Figure 7B and Supplementary Figure 15). The log EC_50_ values for phenylephrine-stimulated vasoconstriction does not differ between the three groups (sham = −6.05 ± 0.16, *n* = 5; SAH = −6.33 ± 0.15, *n* = 5; SAH + terazosin = −6.09 ± 0.15, *n* = 6; P = N.S. by ANOVA). As expected, terazosin treatment has no effect on myogenic reactivity in olfactory cerebral arteries isolated from sham mice (Supplementary Figure 16).

We complemented these experiments by assessing myogenic reactivity in olfactory cerebral arteries isolated from smooth muscle cell-targeted Gq_11_ knockout mice. As in cremaster arteries (Figure 4D), the loss of Gq_11_ expression abolishes phenylephrine-stimulated vasoconstriction in olfactory cerebral arteries (Figure 7C). However, in contrast to cremaster arteries (Figure 4E), olfactory artery myogenic reactivity is not affected by the smooth muscle cell-targeted loss of Gq_11_ expression (Figure 7D). This is consistent with our conclusion that α-adrenergic signaling does not modulate persistent effects on vascular tone in cerebral arteries, in contrast to skeletal muscle resistance arteries.

## Discussion

Since effectively managing blood pressure is a key aspect of SAH therapy, understanding the peripheral cardiovascular effects of SAH is clinically relevant. To this end, our investigation examined the effects of elevated catecholamines on cardiac function and vascular reactivity in an experimental mouse model of SAH. We show that SAH reduces cardiac output and elevates total peripheral resistance *in vivo*; in isolated skeletal muscle resistance arteries *ex vivo*, SAH augments myogenic vasoconstriction. These cardiac and vascular effects are driven by beta- and alpha-adrenergic receptors, respectively. Therapeutically targeting adrenergic receptors in SAH patients, therefore, may possess utility in managing the peripheral effects of SAH during recovery.

Because SAH is a highly-complex pathology, there are no experimental SAH models that perfectly recapitulate all of the clinical aspects of the disease. The blood injection model utilized in the present study has several key advantages, including a relatively uniform hemorrhage/injury stimulus, low mortality and technical ease; however, the model does not simulate an aneurysmal rupture (i.e., a punctured artery) and does not fully mimic the changes in intracranial pressure that occur clinically as a result of subarachnoid bleeding (Leclerc et al., 2018). Further, rodents eliminate blood within the subarachnoid space much faster than humans, resulting in a species-specific time course for the pathology (Lin et al., 2003). Consequently, we (Yagi et al., 2015; Lidington et al., 2019) and others (Lin et al., 2003) have demonstrated that cerebrovascular dysfunction and secondary brain injury develop much more rapidly in experimental rodent models, than what is clinically observed in humans (Macdonald, 2014; Leclerc et al., 2018). For example, cerebral vasospasm (i.e., constriction of large cerebral arteries) occurs within hours to 5 days post-SAH in experimental models, versus 3–12 days post-SAH in patients (Macdonald, 2014; Leclerc et al., 2018). With this in mind, the present study set the assessment timepoint at 2-days post-SAH induction, which aligns with SAH’s peak effect on myogenic tone in cerebral arteries (Yagi et al., 2015) and is a key therapeutic window for intervention in the model (Yagi et al., 2015; Lidington et al., 2019).

To our knowledge, this study is the first to demonstrate secondary cardiac injury in a blood-injection model of SAH. Clinically, 16–30% of patients with SAH experience left ventricular dysfunction within the first 2 days of SAH (Banki et al., 2006; Kerro et al., 2017). The cardiac phenotype that we observe in our experimental model, ventricular dysfunction with normal ejection fraction, occurs in approximately half of the patients that develop SAH-induced neurogenic cardiomyopathy (Banki et al., 2006). Although cardiac dysfunction in SAH is widely attributed to catecholamine toxicity (Wybraniec et al., 2014; Kerro et al., 2017), there is a distinct lack of clinical data to guide intervention: consequently, the treatment of neurocardiac injury in SAH remains largely empirical. However, catecholamine toxicity is not unique to SAH: indeed, elevated catecholamines are widely known to drive profound cardiac injury in a number of disease entities, most notably heart failure (Lymperopoulos et al., 2013). In this regard, there is a wealth of clinical data demonstrating that β-blockers protect the heart against catecholamine-derived injury (Lymperopoulos et al., 2013). Yet, β-blockade in SAH has yielded mixed results: while a few studies purport a beneficial, cardioprotective effect (Neil-Dwyer et al., 1985; Liang et al., 2013), most other retrospective studies find no concrete evidence that taking β-blockers prior to SAH protects against neurocardiac injury (Malik et al., 2015; Luo et al., 2017).

Consistent with the accepted catecholamine toxicity paradigm (Wybraniec et al., 2014; Kerro et al., 2017), cardiac dysfunction is fully prevented by the β-adrenergic receptor antagonist bisoprolol. On the surface, our data appear to contradict the negative clinical analyses; however, there are two significant confounds that likely contribute to the discrepancy: (i) patients on β-blockers prior to SAH either already have cardiac injury or are highly vulnerable to cardiac injury, rendering direct comparisons to a non-β-blocker control group difficult; and (ii) the β-blocker dosage in patients is titrated with respect to their basal sympathetic activity and may be overwhelmed by the SAH-induced catecholamine surge. Indeed, our preliminary experiments indicated that a pre-treatment approach is required for efficacy: since the catecholamine surge is highest immediately following aneurysmal rupture, it is entirely reasonable that effective treatment requires adequate β-blockade at the time of SAH. Our pre-treatment requirement provides additional insight, in that it suggests that the persistently elevated catecholamines that follow the initial surge do not maintain or substantively aggravate the cardiac dysfunction induced by the surge itself. Taken together, our data indicate that β-blockers are cardioprotective in SAH, but that the therapeutic time window is very narrow and therefore, cardioprotection may be clinically difficult to achieve.

Intuitively, persistently high sympathetic activity and circulating catecholamines must alter microvascular function; yet, no studies to date have assessed peripheral vascular function in SAH. In this investigation, we measured skeletal muscle resistance artery myogenic tone, because: (i) myogenic reactivity is a key determinant of microvascular resistance; and (ii) skeletal muscle resistance arteries form the body’s largest circulatory network and therefore, prominently contribute to total peripheral resistance (Korthuis, 2011). Our myogenic tone measurements are conducted *ex vivo*, in order to eliminate the direct influences of circulating catecholamines and neuronal inputs: our control experiments demonstrating that bisoprolol and terazosin treatment *in vitro* do not impact myogenic tone effectively confirm that the target adrenergic receptors were not activated during *ex vivo* testing. Thus, the effects of SAH and *in vivo* treatments are *persistent* effects on smooth muscle cell function that remain in the absence of continuous adrenergic receptor activation.

Our data demonstrate that SAH induces a persistent augmentation of myogenic tone that correlates with elevated total peripheral resistance *in vivo*. Unlike the cardiac phenotype, this vascular phenotype is not driven by β-adrenergic signaling. Selective β-adrenergic activation *in vivo* and *in vitro* attenuates myogenic vasoconstriction, presumably by stimulating long-lasting vasodilatory mechanisms via endothelial (e.g., nitric oxide) and smooth muscle cell-dependent mechanisms (Guimarães and Moura, 2001). In cremaster skeletal muscle resistance arteries, the most abundant adrenergic receptor subtype is the α_1_-adrenergic receptor (Korthuis, 2011): consequently, pro-constrictive α-adrenergic signaling overwhelms any vasodilative β-adrenergic receptor activity in response to high catecholamine concentrations. Given its abundant receptor expression, it is entirely reasonable that α-adrenergic signaling would drive the augmented myogenic tone phenotype in SAH.

Although myogenic tone and TPR are elevated in our SAH model, the magnitude is not large enough to elicit a hypertensive phenotype. Indeed, the lack of hypertension in our model is surprising, given that it is a prevalent clinical feature of SAH (Qureshi et al., 2007). Since cardiomyopathy is far more prevalent in our experimental model than the 16–30% observed clinically (Banki et al., 2006; Kerro et al., 2017), it is tempting to speculate that the cardiac phenotype in our experimental model effectively masks a mechanism that otherwise would elevate blood pressure in a clinical setting. Accordingly, if the microvascular phenotype is similarly prevalent in both mice and humans, then the lower incidence of cardiac dysfunction in human patients would set the stage for the broadly observed hypertensive phenotype. Translational studies, therefore, are necessary to ascertain whether this vascular phenotype is present clinically in SAH patients.

Although bisoprolol fails as an intervention unless it is used as a pre-treatment, terazosin effectively normalizes vascular reactivity when administered following SAH induction: this indicates that terazosin is more amenable for use as a clinical intervention. Should translational studies observe a catecholamine-dependent augmentation of myogenic tone in arteries derived from human SAH patients, our study predicts that (i) α_1_-adrenergic receptor antagonists may be more effective at lowering blood pressure than other routinely utilized medications and (ii) prophylactic α_1_-adrenergic antagonism in high-risk SAH patients may be an effective approach to prevent delayed secondary increases in blood pressure.

At a mechanistic level, catecholamines and synthetic adrenergic agonists are known to increase vascular smooth muscle cell calcium sensitivity (i.e., the response magnitude elicited by a given cytosolic calcium concentration) (Nishimura et al., 1989; Wier and Morgan, 2003). We therefore hypothesized that SAH augments myogenic reactivity by a calcium sensitization mechanism. Consistent with this hypothesis, phenylephrine treatment *in vitro* augmented both cremaster skeletal muscle resistance artery calcium sensitivity and myogenic tone. Interestingly, this calcium-sensitizing effect is not a universal feature of all pro-constrictive G-protein coupled receptor stimuli, as *in vitro* angiotensin II treatment fails to mimic these phenylephrine effects, despite eliciting vasoconstriction.

Since calcium sensitivity appears to be maximal under sham/control settings, phenylephrine can only increase calcium sensitivity following prolonged withdrawal of the physiological inputs. To further explore this withdrawal aspect, we assessed myogenic reactivity in cremaster arteries isolated from smooth muscle cell-targeted Gq_11_ knockout mice, where α_1_-adrenergic and other pro-constrictive G-protein coupled receptor inputs would be eliminated in vascular smooth muscle cells. Consistent with our *in vitro* treatment data, myogenic tone is strongly attenuated in arteries isolated from smooth muscle cell-targeted Gq_11_ knockout mice, relative to wild-type controls. Olfactory cerebral artery myogenic tone is not affected by Gq_11_ gene deletion, indicating that Gq_11_-dependent/α-adrenergic signals likely do not modulate calcium sensitivity in all vascular beds. This variation is presumably dictated by the distinct differentiation states of the vascular smooth muscle cells within the two respective vascular beds.

Although α-adrenergic signaling is capable of modulating calcium sensitivity in cremaster skeletal muscle resistance arteries, several lines of compelling evidence indicate that this mechanism *does not* mediate the SAH-induced augmentation of myogenic tone: (i) calcium sensitivity appears to be maximal under sham conditions and does not increase in SAH; (ii) terazosin treatment in SAH mice normalizes cremaster artery myogenic tone, but does not alter calcium sensitivity; and (iii) the SAH-induced augmentation in myogenic tone is sensitive to TNF gene deletion, whereas phenylephrine-stimulated calcium sensitization *in vitro* is not. Thus, we rejected our hypothesis that SAH augments myogenic reactivity by a calcium sensitization mechanism and shifted our investigative attention away from mechanisms that modulate calcium sensitivity.

As an important caveat, we have not directly measured intracellular calcium levels in cremaster arteries with a calcium sensitive dye, due to a poor signal-to-noise ratio [i.e., a 50% vasoconstriction in response to phenylephrine stimulates only a 5% change in the calcium-sensitive Fura-2 ratio (Kroetsch et al., 2017)]. As a consequence, this study does not directly define the relationship between intracellular calcium and vasoconstriction. We overcame our limited ability to directly measure calcium by using a calcium ramp under depolarizing conditions, with the presumptions that (i) the opening of L-type calcium channels consistently clamps intracellular calcium in all treatment groups and (ii) the depolarizing condition itself does not obscure differences in calcium sensitivity. Thus, the conclusions drawn from our calcium sensitivity data must be viewed with appropriate caution.

We shifted our focus to TNF, a critical myogenic mechanosensor in cremaster skeletal muscle resistance arteries (Kroetsch et al., 2017). As a mechanosensor, the full-length, membrane-bound form of TNF (mTNF) creates a mechanosensitive pair with its receptors that transmits an outside-in signal through mTNF itself. This is termed a “reverse signal,” because the canonical signaling mechanism involves the cleavage-based release of a soluble TNF form that signals as a receptor ligand (Horiuchi et al., 2010; Kroetsch et al., 2017). In mouse cremaster arteries, acutely deleting the TNF gene or scavenging TNF with etanercept strongly attenuates myogenic responsiveness (Kroetsch et al., 2017), suggesting that it plays a dominant role in the mechanotransduction process.

Three important aspects about mTNF mechanosensor function must be highlighted: first, not all vascular beds utilize mTNF as a mechanosensor. In cerebral arteries, for example, TNF only signals via the canonical receptor ligand mechanism (Yang et al., 2012; Yagi et al., 2015; Kroetsch et al., 2017). Second, due to the physiological importance of myogenic reactivity, a compensatory mechanosensor mechanism engages in cremaster arteries when TNF is genetically deleted. We have not precisely defined the nature of this compensatory mechanism; however, it is clear that while myogenic reactivity is restored, functions unique to TNF are not (Kroetsch et al., 2017). In the present study, both of our TNF knockout models had ostensibly engaged this compensatory mechanism and thus, possessed myogenic tone despite the loss of mTNF signaling. Finally, acute TNF inhibiton does not completely abolish pressure-stimulated calcium elevations (Kroetsch et al., 2017), suggesting the presence of other mechanosensitive elements. Although our previous work indicates a substantive role for mTNF in mechanotransduction, it is unlikely to be the sole contributor. Indeed, the compensatory mechanism that emerges following TNF gene deletion may reflect the upregulation of minority contributor to myogenic reactivity.

In the present study, both germline and smooth muscle cell-targeted TNF gene deletion abolishes the SAH-induced augmentation of myogenic tone. Since soluble TNF *does not* augment myogenic reactivity in cremaster arteries (Kroetsch et al., 2017), we propose that α-adrenergic signaling in SAH enhances reverse signals that are mechanically initiated by mTNF. This conclusion is consistent with our observations that terazosin treatment fails to normalize the augmented myogenic tone in cerebral arteries that (i) do not use mTNF as a myogenic mechanosensor (Kroetsch et al., 2017) and (ii) augment myogenic tone via the canonical soluble TNF/TNF receptor-dependent mechanism (Yagi et al., 2015). Unfortunately, we are unable to mechanistically define the nature of the altered mTNF signaling for two important reasons: (i) TNF protein expression analyses are inherently difficult in cremaster arteries, because TNF is not an abundant protein and the tissue sample size is very small; and (ii) because the mTNF reverse signaling field is still in its infancy (Vudattu et al., 2005; Kisiswa et al., 2013), the identities of the cofactors that mediate mTNF reverse signaling are not currently characterized. With regard to the former, since cremaster artery mRNA expression does not change in SAH, we speculate that α-adrenergic signaling changes either the expression or activity of signaling elements that co-localize with mTNF and transduce the reverse signal, rather than changing the expression of TNF itself. The latter issue is beyond the scope of the present investigation, but represents an intriguing investigative direction to follow.

In summary, cardiac injury and spontaneous secondary hypertension are clearly associated with poor clinical outcomes in SAH: this investigation provides evidence for potential cardioprotective interventions and establishes new leads with respect to the putative cause of secondary hypertension in SAH. Our study shows that clinically utilized adrenergic receptor antagonists can prevent cardiac injury and normalize vascular function. With respect to the latter, our data suggest that α-adrenergic signaling augments mTNF-dependent mechanosensor function in skeletal muscle resistance arteries: future work targeting this mechanism could uncover new and valuable therapeutic targets to manage peripheral vascular reactivity and resistance in SAH patients.

## Data Availability Statement

All datasets generated for this study are included in the article/Supplementary Material.

## Ethics Statement

The animal study was reviewed and approved by Animal Care and Use Committee, University of Toronto.

## Author Contributions

DD, JK, DL, and S-SB conceived and designed the experiments. SN, SH, and S-SB contributed to the critical expertise, materials, and techniques. DD, JK, CN, and HZ performed the experiments. DD, JK, DL, CN, HZ, and S-SB analyzed the data. DL wrote the manuscript. All authors reviewed and edited the final manuscript.

## Conflict of Interest

The authors declare that the research was conducted in the absence of any commercial or financial relationships that could be construed as a potential conflict of interest.
