# Supplementary material for: Experimental Subarachnoid Hemorrhage Drives Catecholamine-Dependent Cardiac and Peripheral Microvascular Dysfunction
Source: Front Physiol. 2020 May 13;11:402. doi: 10.3389/fphys.2020.00402 (PMC7237757; doi:10.3389/fphys.2020.00402)
Supplement: Supplementary file 1 [file Presentation_1.pdf]

SUPPLEMENTARY FIGURE 1

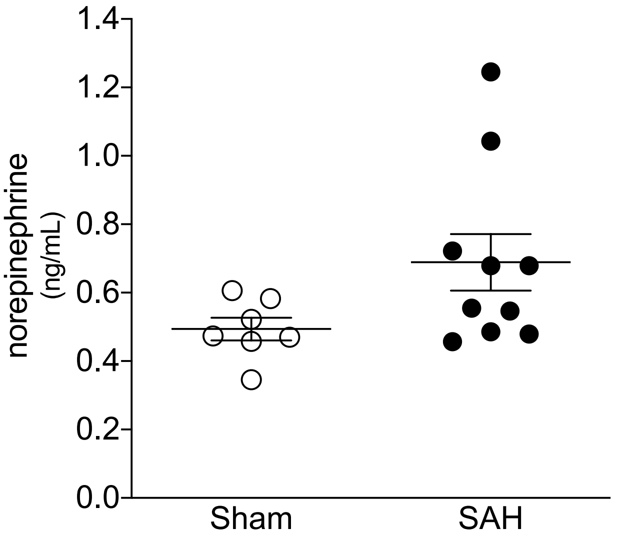

Plasma norepinephrine levels mice with subarachnoid hemorrhage

At 2-days post-subarachnoid hemorrhage (SAH) induction, circulating plasma norepinephrine levels are not different between SAH and sham-operated control mice (sham n=7, SAH n=10). The SAH group did not pass the Shapiro-Wilk normalicy test; the data are therefore compared with a non-parametric Mann-Whitney test (P=NS).

SUPPLEMENTARY FIGURE 2

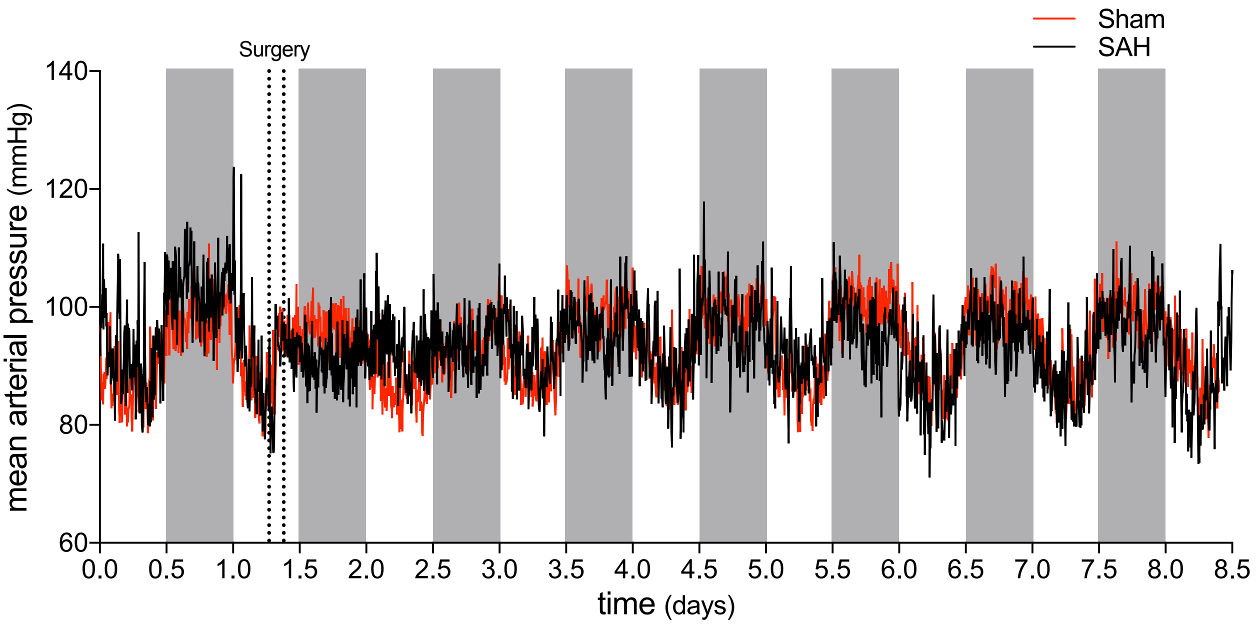

Telemetric blood pressure measurements in mice with subarachnoid hemorrhage

Telemetric mean arterial blood pressure measurements in conscious mice indicate that mice with subarachnoid hemorrhage (SAH; n=3) *do not* develop hypertension within 1-week post-SAH induction (sham n=5). Baseline measurements were collected 1 day prior to SAH or sham surgery; the surgical window is delineated by dotted vertical lines. White and gray shading indicate "lights on" and "lights off" periods, respectively.

SUPPLEMENTARY FIGURE 3

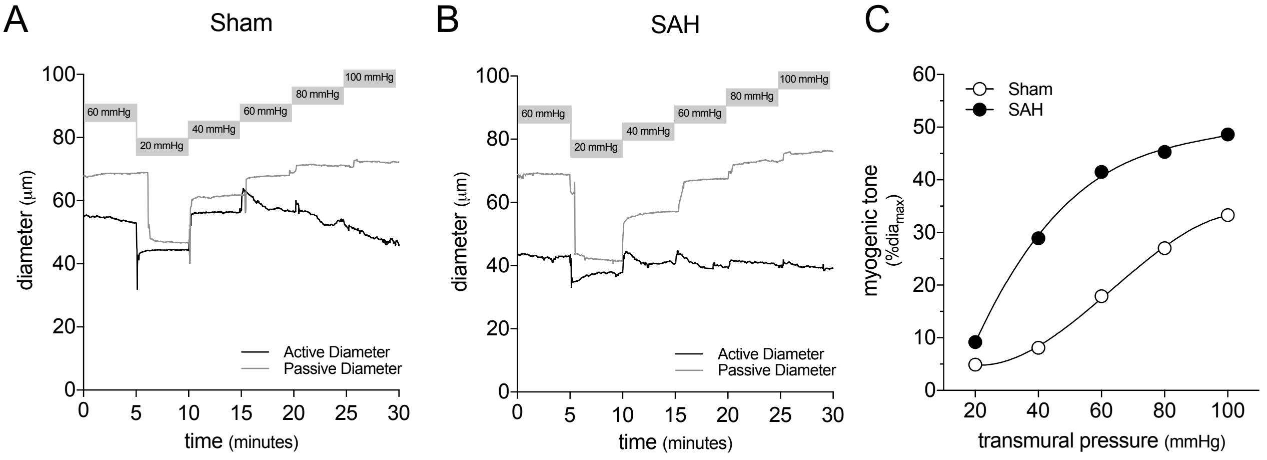

Representative diameter measurements

Stepwise increases in transmural pressure (20-100mmHg) are indicated in the boxes above the tracing. The active diameter tracing is acquired first (“dia_{active}”; black line), followed by measurement of passive diameter under calcium-free conditions (“dia_{max}”; gray line). Shown are representative diameter tracings from cremaster skeletal muscle resistance arteries isolated from **(A)** sham-operated mice ($dia_{max} = 68$ µm) and **(B)** mice with subarachnoid hemorrhage (SAH; $dia_{max} = 68$ µm). Myogenic tone is calculated as the percent constriction in relation to the maximal diameter at each respective transmural pressure: tone (% of dia_{max}) = $[(dia_{max}-dia_{active})/dia_{max}]x100$. ***Panel C*** displays the calculated myogenic tone measures for the arteries displayed in *Panels A and B*.

SUPPLEMENTARY FIGURE 4

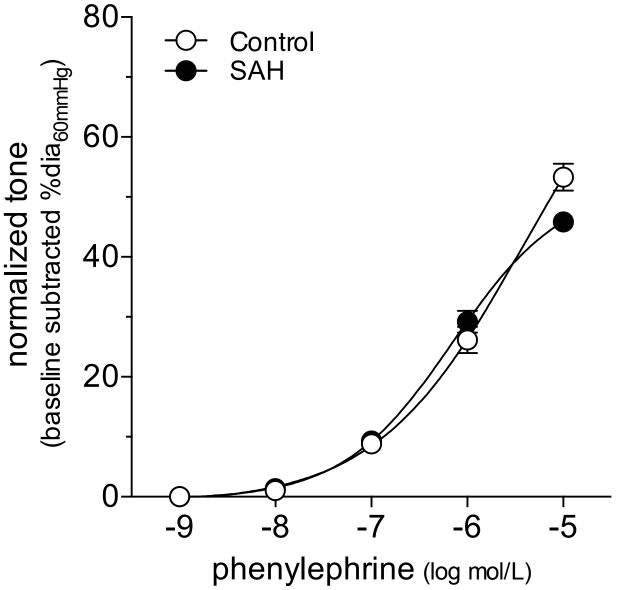

Baseline-normalized phenylephrine responses mice with subarachnoid hemorrhage

When baseline tone is normalized, phenylephrine-stimulated vasoconstriction in cremaster skeletal muscle resistance arteries isolated from mice with subarachnoid hemorrhage (SAH; at 2 days post-induction) are not different from sham-operated controls. Data were statistically compared with a 2-way ANOVA (P=N.S.; sham dia_{max}: 78±2 µm, n=26; SAH 78±2 µm, n=22).

SUPPLEMENTARY FIGURE 5

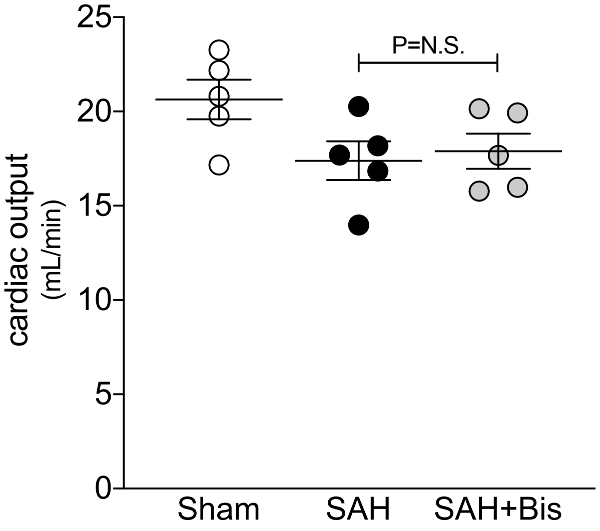

Post-operative bisoprolol treatment does not protect cardiac function following subarachnoid hemorrhage

When delivered immediately following experimental subarachnoid hemorrhage (SAH) induction, bisoprolol treatment (Bis; twice daily for 2 days with i.p. injections; 10 mg/kg initial pre-operative dose followed by 5 mg/kg for all subsequent injections) does not prevent the reduction in cardiac output at 2-days post-SAH (n=5 for all groups). The SAH and SAH+Bis groups were statistically compared with a Student's t test (P=N.S.).

SUPPLEMENTARY FIGURE 6

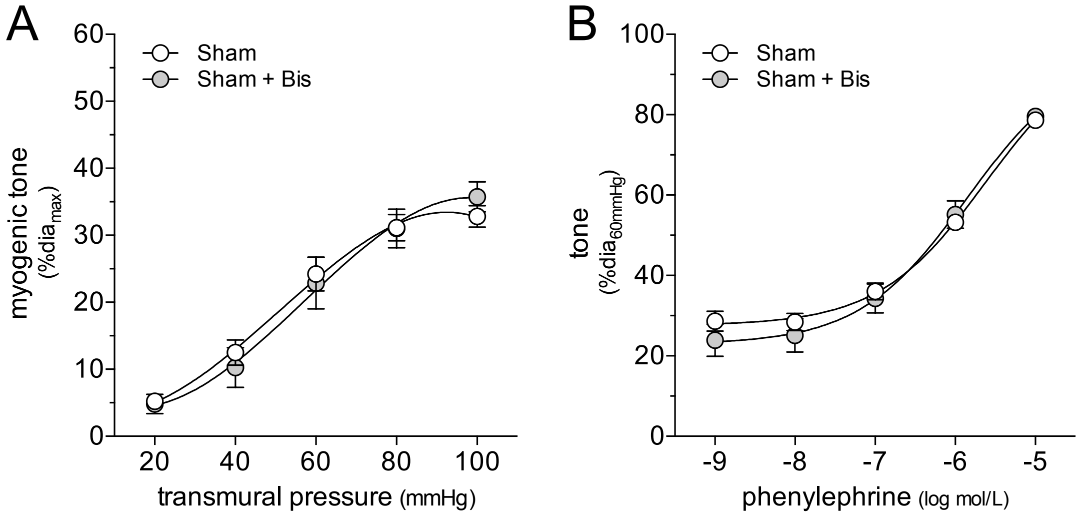

Bisoprolol does not alter cremaster artery reactivity in sham animals

In sham-operated mice, *in vivo* bisoprolol treatment (Bis; twice daily for 2 days with i.p. injections; 10 mg/kg initial pre-operative dose followed by 5 mg/kg for all subsequent injections) has no effect on **(A)** myogenic reactivity or **(B)** phenylephrine responses in cremaster skeletal muscle resistance arteries isolated at 2 days post-surgery/treatment (sham dia_{max}: 80±2 µm, n=13; Sham+Bis dia_{max}: 70±4 µm, n=9). The sham data in both panels are reproduced from *Figure 2* for comparison to the Sham+Bis data. Data were statistically compared with a 2-way ANOVA (P=N.S.)

SUPPLEMENTARY FIGURE 7

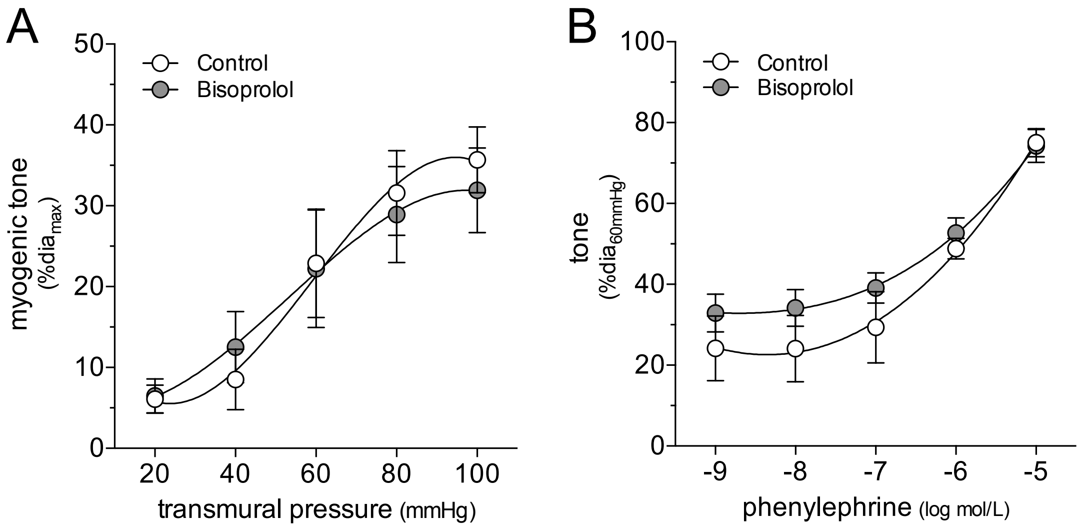

Bisoprolol does not alter cremaster artery reactivity in vitro

In vitro, bisoprolol (5 µmol/L for 30 minutes) does not affect cremaster skeletal muscle resistance artery **(A)** myogenic reactivity or **(B)** phenylephrine responses (dia_{max} = 76±5, n=4). Data were statistically compared with a repeated measures 2-way ANOVA (P=N.S.).

SUPPLEMENTARY FIGURE 8

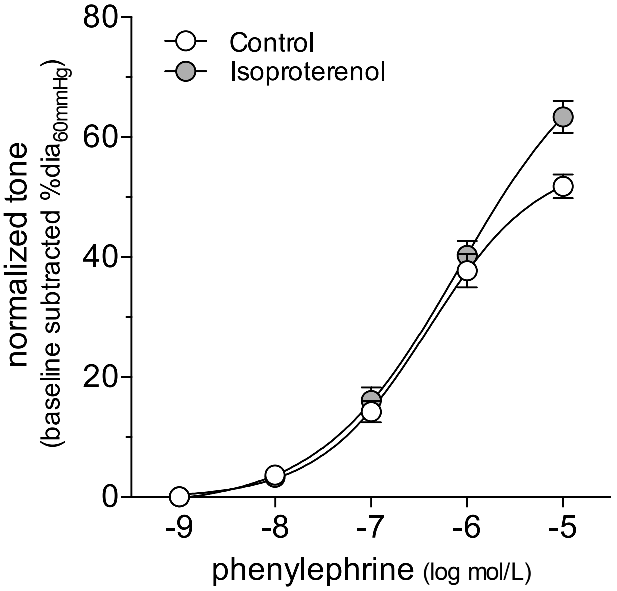

Baseline-normalized phenylephrine responses for isoproterenol-treated mice

When baseline tone is normalized, phenylephrine responses in cremaster skeletal muscle resistance arteries isolated from isoproterenol-treated mice (150 mg/kg/day i.p. for 2 days) are not different from saline-treated controls. Data were statistically compared with a 2-way ANOVA (P=N.S.; control dia_{max}: 80±4 µm, n=8; isoproterenol dia_{max}: 79±2 µm, n=8).

SUPPLEMENTARY FIGURE 9

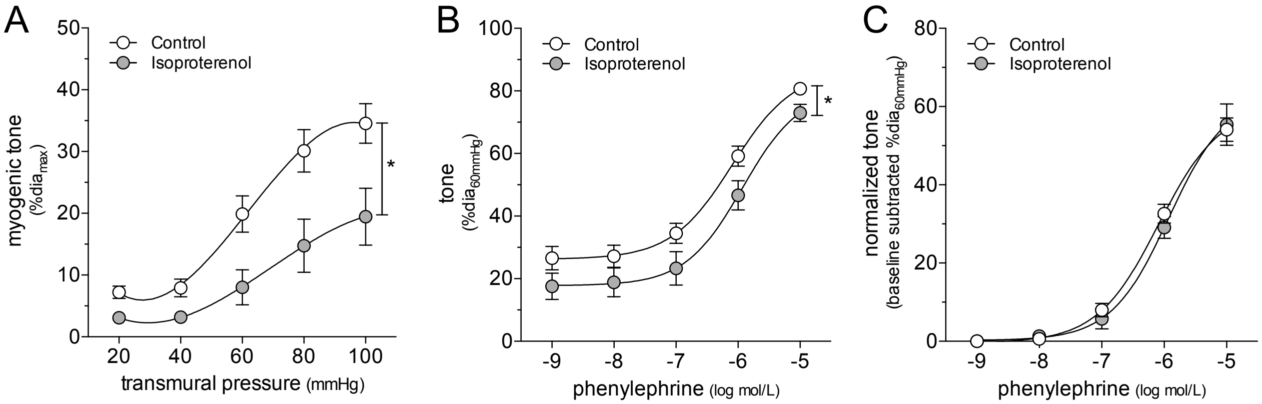

Isoproterenol alters cremaster artery myogenic reactivity in vitro

(A) *In vitro*, isoproterenol (1µmol/L for 30 minutes) attenuates cremaster skeletal muscle resistance artery myogenic reactivity. **(B)** Phenylephrine responses are also attenuated by isoproterenol treatment, due to a shift in basal tone. **(C)** When baseline tone is normalized, phenylephrine responses are unaffected by isoproterenol treatment. All data were statistically compared with a paired 2-way ANOVA (dia_{max} = 73±3, n=8). * denotes $P<0.05$.

SUPPLEMENTARY FIGURE 10

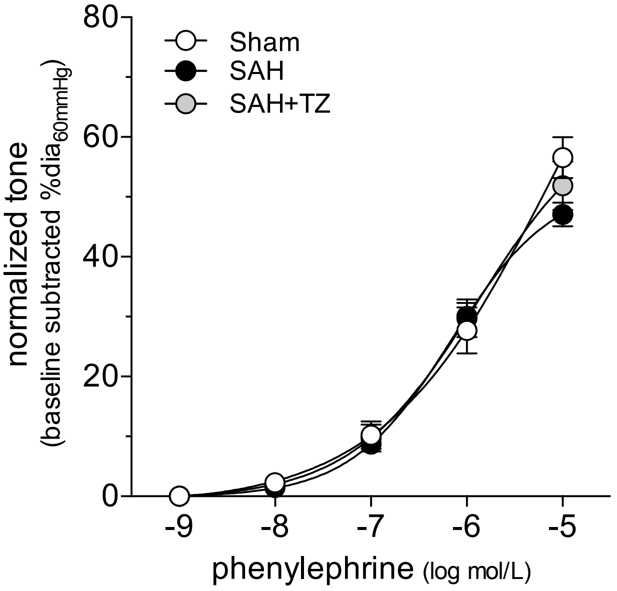

Baseline-normalized phenylephrine responses for terazosin-treated mice

When baseline tone is normalized, phenylephrine responses in cremaster skeletal muscle resistance arteries isolated from SAH mice are not different from those isolated from sham-operated controls. Normalized phenylephrine responses in cremaster skeletal muscle resistance arteries isolated from terazosin-treated mice (TZ; twice daily for 2 days with i.p. injections; 1 mg/kg initial pre-operative dose followed by 0.5 mg/kg for all subsequent injections) are also not different from sham-operated controls. Data were statistically compared with a 2-way ANOVA (P=N.S.; sham dia_{max}: 76±3 µm, n=13; SAH dia_{max}: 76±3 µm, n=12; SAH+TZ dia_{max}: 74±2 µm, n=13).

SUPPLEMENTARY FIGURE 11

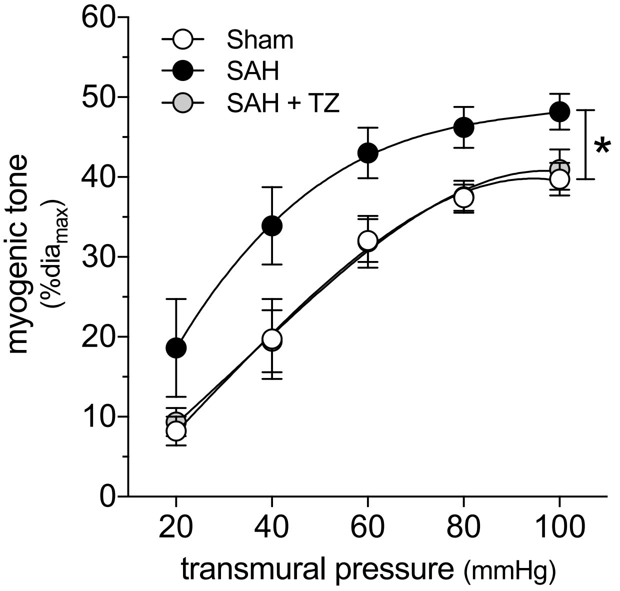

Post-operative terazosin treatment normalizes myogenic reactivity following subarachnoid hemorrhage

When delivered immediately following experimental subarachnoid hemorrhage (SAH) induction, terazosin (TZ; twice daily for 2 days with i.p. injections; 1 mg/kg initial dose followed by 0.5 mg/kg for all subsequent injections) normalizes the augmented myogenic vasoconstriction observed in cremaster skeletal muscle resistance arteries at 2-days post-SAH. Data were statistically compared with a 2-way ANOVA (sham dia$_{max}$: 72±4 µm, n=5; SAH dia$_{max}$: 76±6 µm, n=5; SAH+TZ dia$_{max}$: 72±5 µm, n=6). * denotes $P<0.05$.

SUPPLEMENTARY FIGURE 12

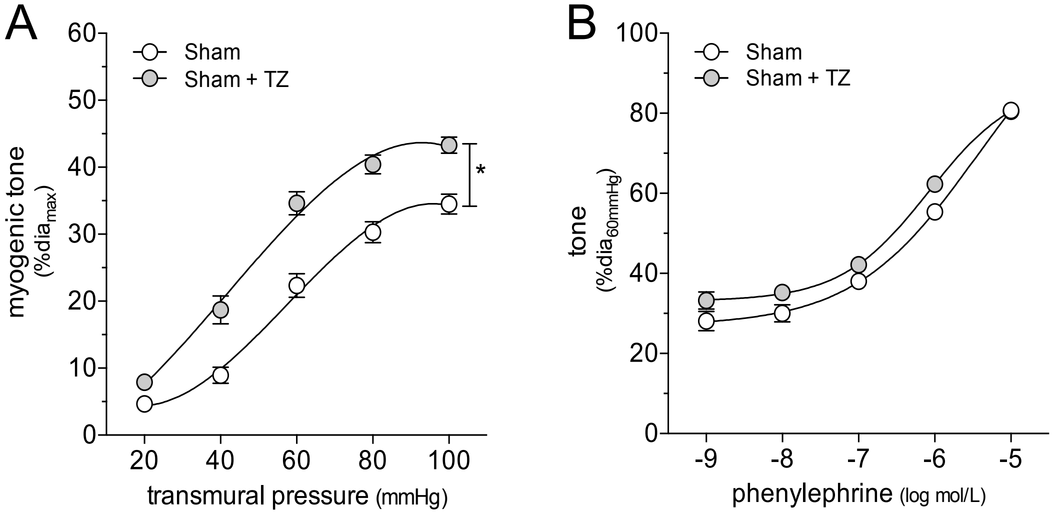

Terazosin augments cremaster artery myogenic reactivity in sham animals

In sham-operated mice, *in vivo* terazosin treatment (TZ; twice daily for 2 days with i.p. injections; 1 mg/kg initial pre-operative dose followed by 0.5 mg/kg for all subsequent injections) **(A)** augments myogenic reactivity in cremaster skeletal muscle resistance arteries isolated at 2 days post-surgery/treatment, but **(B)** does not alter phenylephrine responses (sham dia_{max}: 75±2 µm, n=19; Sham+TZ dia_{max}: 76±3 µm, n=26). * denotes $P<0.05$.

SUPPLEMENTARY FIGURE 13

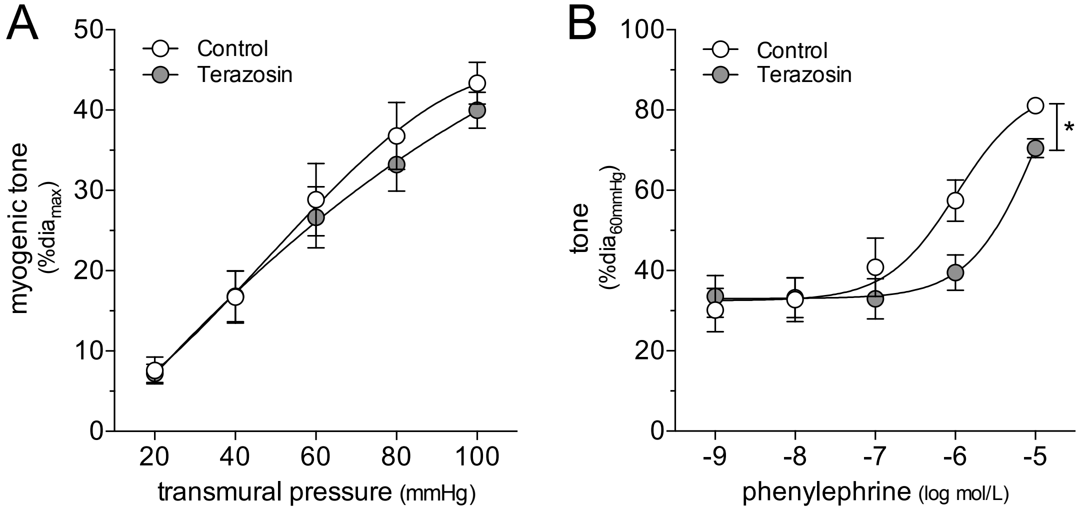

Terazosin does not alter cremaster artery myogenic reactivity in vitro

(A) *In vitro*, terazosin (25nmol/L for 30 minutes) has no effect on cremaster skeletal muscle resistance artery myogenic reactivity. **(B)** However, *in vitro* terazosin treatment attenuates phenylephrine-stimulated vasoconstriction. Data were statistically compared with a repeated measures 2-way ANOVA (dia_{max} = 78±4, n=6). * denotes P<0.05.

SUPPLEMENTARY FIGURE 14

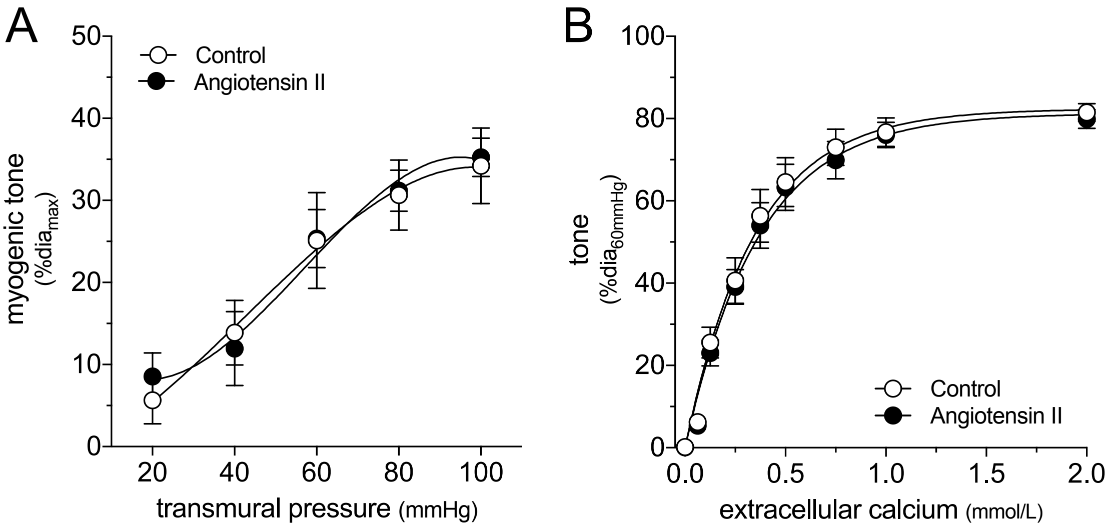

In vitro angiotensin II treatment does not alter cremaster artery myogenic reactivity or calcium sensitivity

Cremaster skeletal muscle resistance arteries isolated from naïve mice were treated for 4 hours with either 10 nmol/L angiotensin II or control buffer *in vitro*. The arteries were then washed and assessed in normal buffer. Angiotensin treatment does not affect **(A)** myogenic tone or **(B)** calcium sensitivity (control dia_{max}: 69±4 µm, n=5; angiotensin II dia_{max}: 78±5 µm, n=6). Data were statistically compared with a 2-way ANOVA.

Supplementary Material

SUPPLEMENTARY FIGURE 15

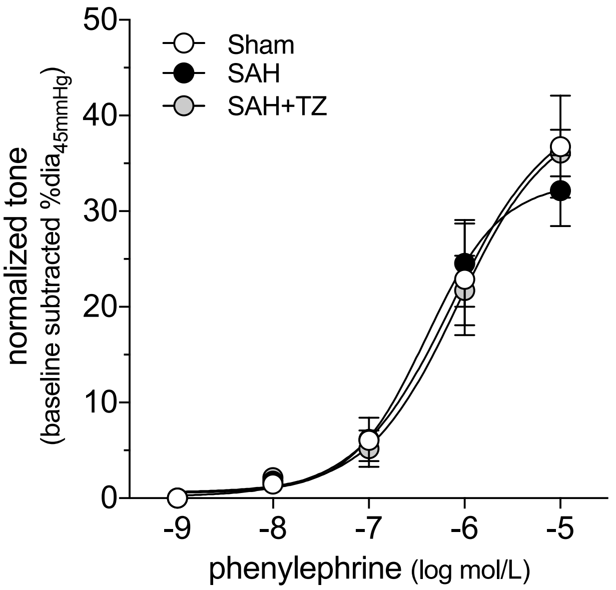

Baseline-normalized phenylephrine responses in terazosin-treated cerebral arteries

When baseline tone is normalized, phenylephrine responses in olfactory cerebral arteries isolated from SAH mice are not different from those isolated from sham-operated controls. Normalized phenylephrine responses in olfactory cerebral arteries isolated from terazosin-treated mice (TZ; twice daily for 2 days with i.p. injections; 1 mg/kg initial pre-operative dose followed by 0.5 mg/kg for all subsequent injections) are also not different from sham-operated controls. Data were statistically compared with a 2-way ANOVA (P=N.S.; sham dia_{max}: 108±4 µm, n=6, SAH dia_{max}: 104±7 µm, n=5, SAH+TZ dia_{max}: 108±10 µm, n=6).

SUPPLEMENTARY FIGURE 16

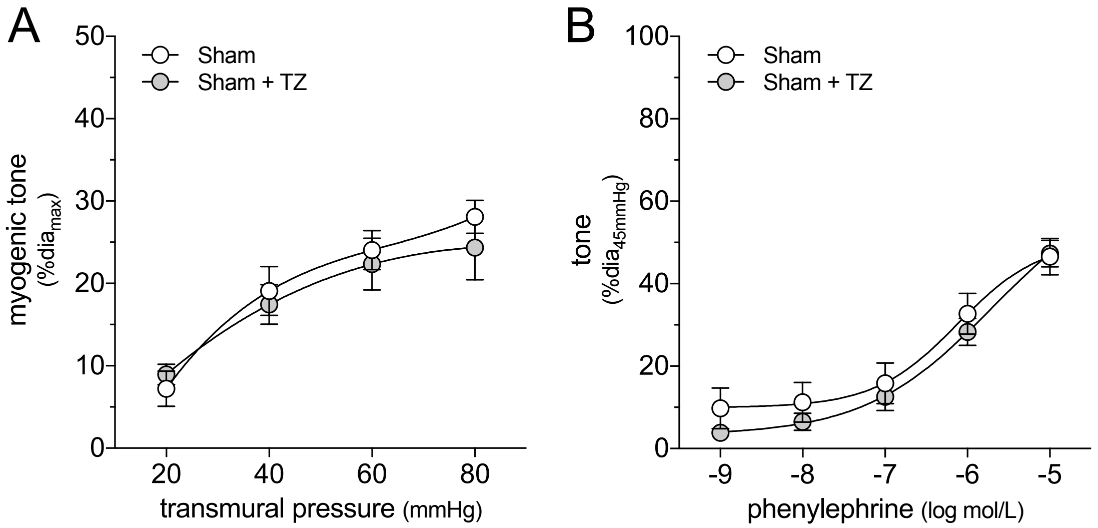

Terazosin does not alter olfactory cerebral artery reactivity in sham animals.

In sham-operated mice, *in vivo* terazosin treatment (TZ; twice daily for 2 days with i.p. injections; 1 mg/kg initial pre-operative dose followed by 0.5 mg/kg for all subsequent injections) has no effect on **(A)** myogenic reactivity (sham dia$_{max}$: 108±4 µm, n=6; Sham+TZ dia$_{max}$: 110±5 µm, n=5) or **(B)** phenylephrine responses (sham dia$_{max}$: 110±4 µm, n=5; Sham+TZ dia$_{max}$: 110±5 µm, n=5) in olfactory cerebral arteries isolated at 2 days post-surgery/treatment. The sham data in both panels are reproduced from *Figure 6*, for comparison to the Sham+TZ data. Data were statistically compared with a 2-way ANOVA (P=N.S.).

Supplementary Material

SUPPLEMENTARY TABLE 1

Echocardiographic measures in mice with subarachnoid hemorrhage

	Sham	SAH
**Mouse Body Weight (g)**	22.9 ± 0.3	22.5 ± 0.3
**Wall Thickness (mm)**	0.658 ± 0.016	0.654 ± 0.022
**Systolic Diameter (mm)**	2.66 ± 0.06	2.68 ± 0.07
**Diastolic Diameter (mm)**	3.87 ± 0.05	3.75 ± 0.06
**Systolic Volume (µl)**	17.8 ± 1.0	18.0 ± 1.1
**Diastolic Volume (µl)**	48.0 ± 1.6	44.2 ± 1.6
**Fractional Shortening (%)**	31.3 ± 0.8	28.9 ± 0.9
**LVEF (%)**	64 ± 1	60 ± 1
**Stroke Volume (µl)**	30.2 ± 0.9	**26.2 ± 0.7 ***
**Heart Rate (min^{-1})**	510 ± 7	**477 ± 10 ***
**Cardiac Output (ml/min)**	15.3 ± 0.4	**12.4 ± 0.4 ***

Data are means ± SEM (n=35 in both groups). * denotes $P<0.05$ for an unpaired comparison (Student's t test). Acronyms: LVEF − Left ventricular ejection fraction; SAH – subarachnoid hemorrhage.

SUPPLEMENTARY TABLE 2

Echocardiographic measures in bisoprolol-treated mice

	Sham	SAH	SAH+Bis
**Mouse Body Weight (g)**	22.3 ± 0.4	21.9 ± 0.5	21.9 ± 0.5
**Wall Thickness (mm)**	0.665 ± 0.020	0.671 ± 0.033	0.653 ± 0.031
**Systolic Diameter (mm)**	2.46 ± 0.05	2.61 ± 0.09	2.49 ± 0.10
**Diastolic Diameter (mm)**	3.68 ± 0.05	3.65 ± 0.08	3.68 ± 0.10
**Systolic Volume (µl)**	15.3 ± 1.4	16.4 ± 1.7	14.2 ± 1.5
**Diastolic Volume (µl)**	41.4 ± 2.1	39.7 ± 2.2	41.1 ± 2.8
**Fractional Shortening (%)**	33.1 ± 1.3	28.4 ± 1.8	32.3 ± 2.1
**LVEF (%)**	64 ± 2	59 ± 3	66 ± 2
**Stroke Volume (µl)**	26.1 ± 1.0	23.3 ± 1.3	26.9 ± 1.7
**Heart Rate (min^{-1})**	542 ± 10	**485 ± 15 ***	512 ± 16
**Cardiac Output (ml/min)**	14.2 ± 0.7	**11.4 ± 0.8 ***	14.6 ± 0.8

Data are means ± SEM (sham n=12; SAH n=11; SAH+Bis n=10). * denotes $P<0.05$ for an unpaired comparison to the sham (ANOVA with Dunnett's post-test). Acronyms: Bis: – bisoprolol; LVEF − Left ventricular ejection fraction; SAH – subarachnoid hemorrhage.

SUPPLEMENTARY TABLE 3

Echocardiographic measures in isoproterenol-treated mice

	Naïve	Isoproterenol
**Mouse Body Weight (g)**	25.4 ± 0.5	24.9 ± 0.4
**Wall Thickness (mm)**	0.662 ± 0.033	0.691 ± 0.030
**Systolic Diameter (mm)**	3.07 ± 0.13	2.89 ± 0.99
**Diastolic Diameter (mm)**	4.24 ± 0.11	**3.87 ± 0.08** *
**Systolic Volume (µl)**	23.1 ± 2.6	21.2 ± 1.6
**Diastolic Volume (µl)**	60.5 ± 4.2	**51.7 ± 1.7** *
**Fractional Shortening (%)**	27.7 ± 1.2	25.4 ± 1.5
**LVEF (%)**	62 ± 2	59 ± 3
**Stroke Volume (µl)**	37.4 ± 2.5	**30.5 ± 1.0** *
**Heart Rate (min^{-1})**	477 ± 12	471 ± 11
**Cardiac Output (ml/min)**	17.8 ± 0.9	**14.4 ± 0.6** *

Data are means ± SEM (naïve n=5; isoproterenol n=9). * denotes $P<0.05$ for an unpaired comparison (Student's t test). Acronym: LVEF − Left ventricular ejection fraction.

SUPPLEMENTARY TABLE 4

Echocardiographic measures in terazosin-treated mice

	**Sham**	**SAH**	**SAH+TZ**
**Mouse Body Weight (g)**	23.9 ± 0.4	23.3 ± 0.3	23.8 ± 0.5
**Heart Wall Thickness**	0.669 ± 0.029	0.684 ± 0.031	0.673 ± 0.025
**Systolic Diameter**	2.74 ± 0.09	2.72 ± 0.10	2.80 ± 0.10
**Diastolic Diameter**	3.93 ± 0.08	3.81 ± 0.10	3.82 ± 0.09
**Systolic Volume**	18.2 ± 1.4	18.7 ± 1.8	21.0 ± 1.9
**Diastolic Volume**	50.4 ± 2.2	46.6 ± 2.5	48.5 ± 2.6
**Fractional Shortening**	30.6 ± 1.2	28.8 ± 1.0	26.9 ± 1.2
**LVEF (%)**	64 ± 2	61 ± 2	**57 ± 2 ***
**Stroke volume (µl)**	32.2 ± 1.3	**27.9 ± 1.0 ***	**27.5 ± 1.7 ***
**Heart rate (min^{-1})**	500 ± 9	480 ± 14	**443 ± 16 ***
**Cardiac Output (ml/min)**	16.0 ± 0.6	**13.3 ± 0.4 ***	**12.0 ± 0.3 ***

Data are means ± SEM (sham n=16; SAH n=16; SAH+TZ n=12). * denotes $P<0.05$ for an unpaired comparison to the sham (ANOVA with Dunnett's post-test). Acronyms: TZ: – terazosin; LVEF – Left ventricular ejection fraction; SAH – subarachnoid hemorrhage.
